# Suramin, screened from an approved drug library, inhibits HuR functions and attenuates malignant phenotype of oral cancer cells

**DOI:** 10.1002/cam4.1877

**Published:** 2018-11-18

**Authors:** Wataru Kakuguchi, Takao Nomura, Tetsuya Kitamura, Satoko Otsuguro, Kazuhiro Matsushita, Masahiro Sakaitani, Katsumi Maenaka, Kanchu Tei

**Affiliations:** ^1^ Department of Oral and Maxillofacial Surgery, Faculty of Dental Medicine and Graduate School of Dental Medicine Hokkaido University Sapporo Japan; ^2^ Center for Research and Education on Drug Discovery, Faculty of Pharmaceutical Sciences Hokkaido University Sapporo Japan; ^3^ Department of Oral Pathology and Biology, Faculty of Dental Medicine and Graduate School of Dental Medicine Hokkaido University Sapporo Japan

**Keywords:** AU‐rich elements, drug repositioning, HuR, screening, suramin

## Abstract

AU‐rich elements (ARE) exist in the 3′‐untranslated regions of the mRNA transcribed from cell growth‐related genes such as proto‐oncogenes, cyclin‐related genes, and growth factors. HuR binds and stabilizes ARE‐mRNA. HuR is expressed abundantly in cancer cells and related malignant phenotypes. HuR knockdown attenuates the malignant phenotype of oral cancer cells. In this study, we screened 1570 compounds in the approved drug library by differential scanning fluorimetry (DSF) to discover a HuR‐targeted compound. Firstly, 55 compounds were selected by DSF. Then, 8 compounds that showed a shift in the melting temperature value in a concentration‐dependent manner were selected by DSF. Of them, suramin, an anti‐trypanosomal drug, binds to HuR, exhibiting fast‐on and fast‐off kinetic behavior on surface plasmon resonance (SPR). We confirmed that suramin significantly decreased mRNA and protein expression of cyclin A2 and cyclin B1. The *cyclin A2* and *cyclin B1* mRNAs were destabilized by suramin. Furthermore, the motile and invasive activities of a tongue carcinoma cell line treated with suramin were markedly lower than those of control cells. The above findings suggest that suramin binds to HuR and inhibits its function. We also showed that the anticancer effects of suramin were caused by the inhibition of HuR function, indicating its potential as a novel therapeutic agent in the treatment of oral cancer. Our results suggest that suramin, via its different mechanism, may effectively suppress progressive oral cancer that cannot be controlled using other anticancer agents.

## 1 INTRODUCTION

Regulation of mRNA decay is one of the important steps of gene expression, as with transcription and translation. AU‐rich elements (ARE) are instability elements that are commonly present in the 3′‐untranslated regions (UTR) of mRNA encoding the so‐called early response genes such as proto‐oncogenes, cyclin‐related genes, and growth factors.^1^, ^2^, ^3^ A number of proteins are known to interact with ARE and modulate either the stabilization or destabilization of ARE‐mRNA.^1^, ^2^, ^4^, ^5^

HuR is an RNA‐binding protein, and it stabilizes not only ARE‐mRNA, but also mRNA not containing ARE.^6^, ^7^ HuR has three RNA recognition motifs (RRMs),^8^ which are highly abundant RNA‐binding domains in eukaryotes.^9^ The first two RRMs form a cleft that facilitates binding of mRNA^8^, ^10^, ^11^, ^12^ and they also interact to form a HuR homodimer.^13^ The hinge region between RRM2 and RRM3 contains the nuclear localization signal.^4^ RRM3 is important for stabilizing the RNA‐protein complex and mediating protein‐protein interactions,^12^ such as interactions with the poly‐A tail of target mRNA.^10^ Moreover, it shows terminal adenosyltransferase activity.^14^

HuR stabilizes ARE‐mRNAs involved in multiple biological events such as carcinogenesis, differentiation, and inflammation.^15^, ^16^ HuR is overexpressed in cancers, such as in oral, colon, ovarian, brain, breast, and pancreatic cancers.^17^, ^18^, ^19^, ^20^, ^21^, ^22^, ^23^ In particular, the abundant cytoplasmic expression of HuR is associated with the malignant phenotype of several types of carcinomas.^19^ We previously reported that HuR knockdown attenuated the malignant phenotype by inhibiting the stabilization of ARE‐mRNA. Inhibition of HuR‐ARE interaction can be an attractive strategy in developing new cancer therapeutics.^7^ Recently, RRMs have been considered for drug design approaches.^11^, ^14^ Some compounds have been reported to target RRM1 and RRM2.^13^, ^24^, ^25^ In addition, compounds targeting RRM3 have been reported.^14^, ^24^ However, the clinical application of these compounds as anticancer drugs will require a long time. To shorten the time until the clinical application of HuR‐targeted drugs, drug repositioning from an approved drug is effective.^26^

In the present study, we screened an approved drug library using differential scanning fluorimetry (DSF) to identify a HuR‐targeted compound. We identified suramin as a suitable candidate that bound to HuR and inhibited HuR function in HSC‐3 and SAS cells, a tongue carcinoma cell line. Given its potential to be used clinically, suramin activities were further evaluated in this study.

## 2 MATERIALS AND METHODS

### 2.1 Expression and purification of HuR protein

BL21 competent *Escherichia coli* (C2530H, NEB, Ipswich, MA, USA) cells were transformed with pGEX‐6P‐1 vectors containing sequences coding for full‐length HuR (326 residues) and incubated in Overnight Express^TM^ lnstant LB medium (Merck Millipore, Billerica, MA, USA). *E. coli* was resuspended in Bug Buster Master Mix (Merck Millipore). Insoluble cell debris was removed by centrifugation at 16 000 × *g* for 20 minutes at 4°C. Recombinant GST‐HuR protein was pulled down by Glutathione Sepharose 4B (GST SpinTrap and GSTrap 4B; GE Healthcare, Uppsala, Sweden) column. HuR protein was separated from GST with PreScission Protease (GE Healthcare) and dissolved in HN buffer (20 mmol/L HEPES and 150 mmol/L NaCl buffer at pH 7.4). When the concentration of HuR protein was low, the protein was concentrated with Amicon Ultra centrifugal filters (Merck Millipore). The protein concentration was determined by TaKaRa Bradford Protein Assay Kit (TaKaRa, Kusatsu, Shiga, Japan). The solubility of GST‐HuR, HuR, and GST proteins was confirmed with gel staining and Western blot (Figure S1).

### 2.2 Differential scanning fluorimetry (DSF)

Thermal unfolding of HuR constructs was monitored by DSF in the presence of fluorescent SYPRO Orange dye (Invitrogen, Eugene, OR, USA) by using a CFX96 Real‐Time PCR Detection System (Bio‐Rad, Hercules, CA, USA). An approved compound library, Pharmakon (MicroSource Discovery Systems, Gaylordsville, CT, USA), was used. The compounds were transferred to 96‐well microplates [10 mmol/L, dissolved in dimethyl sulfoxide (DMSO)]. Because 30 compounds of the library could not be imported due to ban on drugs import, DMSO was added to the wells instead of these compounds. HuR protein (5 μg) and the dye in HN buffer were used. Quercetin, which has been reported to bind HuR,^27^ was used as a positive control. DMSO was used as a negative control. The thermal unfolding process was monitored between 20 and 90°C, by increasing the temperature at a rate of 0.5°C. The values of melting temperature (*T*_m_) were calculated. The compounds were used at 200 μmol/L in the final 1% of DMSO for HuR protein (5 μg) in the first DSF and 467 μmol/L in the final 0.47% of DMSO for HuR protein (5 μg) in the second DSF.

### 2.3 Surface plasmon resonance (SPR)

The SPR experiment was performed on a BIACORE T200 instrument (GE Healthcare) equipped with a CM5 sensor chip. The sensor chip was activated via an amine‐coupling reaction with EDC and NHS. Then, HuR protein (36 kD) was applied to a flow cell with 10 mmol/L HEPES buffer at pH 7.0. HuR protein was immobilized at a density of 483 RU (response units). The surface was blocked with 1 mol/L ethanolamine hydrochloride at pH 8.5. To collect kinetic binding data, suramin in 10 mmol/L HEPES and 150 mmol/L NaCl (pH 7.4) was injected into the flow cell at the indicated concentrations at 25°C.

### 2.4 Cells

HSC‐3 and SAS, human tongue squamous cell carcinoma cell lines, were purchased from RIKEN BRC Cell Bank (Tsukuba, Japan) and JCRB Cell Bank (Osaka, Japan), respectively. The cells were cultured at 37°C under a 5% CO_2_ atmosphere in Dulbecco's modified Eagle medium (DMEM, Nissui Pharmaceutical, Ueno, Tokyo, Japan) containing 10% fetal bovine serum (FBS) and penicillin/streptomycin/amphotericin B (Sigma, St Louis, MO, USA).

### 2.5 Western blot analysis

Western blot analysis was performed on cells treated with different concentrations of suramin sodium (suramin) (Wako Pure Chemical Industries, Osaka, Japan) (0, 20, 50, and 100 μmol/L) for 24 hours in DMEM without FBS. The cells were homogenized in RIPA buffer (25 mmol/L Tris‐HCl, pH 7.6, 150 mmol/L NaCl, 1% NP‐40, 1% sodium deoxycholate, and 0.1% SDS) with protease inhibitor cocktail (p8340, Sigma). The protein concentration was determined by the Pierce™ BCA Protein Assay Kit (Thermo Fisher Scientific, Rockford, IL, USA). Samples (20 μg) were separated on 10% TGX FastCast™ acrylamide gel (Bio‐Rad) and transferred to a Amasham™ Hybond™ 0.2 μm polyvinylidene fluoride (PVDF) membrane (GE Healthcare). Blocking of the membrane was performed using PBS with 5% skim milk powder (Wako Pure Chemical Industries). The antibodies used in this study were specific to HuR (sc‐5261, 1:2000, Santa Cruz, Santa Cruz, CA, USA), cyclin A (611268, 1:2000), cyclin B1 (554178, 1:2000), Cox2 (610203, 1:2000, BD Biosciences, Cambridge, MA, USA), and β‐actin (A5441, 1:10 000, Sigma). The secondary antibody was horseradish peroxidase‐conjugated IgG (Jackson ImmunoResearch Laboratories, 1:5000‐10 000, West Grove, PA, USA). The protein bands were visualized with the Amersham™ ECL™ start or Prime Western Blotting Detection Reagent (GE Healthcare) and detected by LAS‐4000 mini (GE Healthcare).

### 2.6 Real‐time quantitative reverse transcription PCR (real‐time qRT‐PCR)

qRT‐PCR was performed on cells treated with different concentrations (0, 20, 50, and 100 μmol/L) of suramin for 24 hours in DMEM without FBS. Total cellular RNA was isolated using TRI reagent (Molecular Research Center, Inc, Montgomery Rd, OH, USA) according to the manufacturer's protocol. Selected total RNA samples (1 μg) were reverse‐transcribed using ReverTra Ace (Toyobo, Osaka, Japan). CFX96 Real‐Time PCR Detection System (Bio‐Rad) and the SSO Advanced™ Universal CYBR Green Supermix (Bio‐Rad) were used for qRT‐PCR. cDNA was amplified using the following primers: for *cyclin A2*: 5′‐AGCTGCCTTTCATTTAGCACTC‐3′, 5′‐TGCTTTGAGGTAGGTCTGGTG‐3′; for *cyclin B1*: 5′‐TGTGGATGCAG ‐AAGATGGAG‐3′, 5′‐AACCGATCAATAATGGAGACAG‐3′; for *c‐fos*: 5′‐CCAACCTGCTGAAGGAGAAG‐3′, 5′‐GCTGCTGATGCTCTTGACAG‐ 3′; for *c‐myc*: 5′‐CTCCTGGCAAAAGGTCAGAG‐3′, 5′‐TCGGTTGTTGCT‐ GATCTGTC‐3′; for *COX‐2*:5′‐TGAGCATCTACGGTTTGCTG‐3′, 5′‐TGC‐ TTGTCTGGAACAACTGC‐3′; for *cdk1*: 5′‐TTCAGGATGTGCTTATGCAG‐ GA‐3′, 5′‐AGTGACAAAACACAATCCCCTGTAG‐3′ and for *GAPDH*: 5′‐ATCCTGGGCTACACTGAGCA‐3′, 5′‐TGCTGTAGCCAAATTCGTTG‐3′. GAPDH was used for normalization. Relative quantification was performed using the 2^−ΔΔ^ *^Ct^* method.

To evaluate the half‐life of total ARE‐mRNA, both control cells and 50 μmol/L suramin‐treated cells were treated with 5 μg/mL actinomycin d‐mannitol (Sigma) for 2 or 4 hours. The RNA extracted after treatment with actinomycin d‐mannitol was subjected to qRT‐PCR.

### 2.7 MTS assay

The CellTiter 96R Aqueous One Solution Cell Proliferation Assay (Promega, Madison, WI, USA) was used to detect the viable cells in the proliferation and cytotoxicity assay. HSC‐3 cells (5 × 10^3^) were harvested in 96‐well plates. After 24 hours, the culture medium was discarded and the cells were washed in phosphate‐buffered saline (PBS). Then, DMEM without FBS containing suramin at various concentrations (0, 5, 10, 20, 50, 75, 100, 200, 400, 600, 800, and 1000 μmol/L) was added to the wells. After 24 hours, MTS reagents were added for 4 hours, and the absorbance at optical density (OD) 490 nm was recorded using a microplate reader (Bio‐Rad).

### 2.8 Wound healing assay

HSC‐3 and SAS cells were wounded with a 200‐μL pipette tip and washed with PBS. The cells were then incubated for 24 hours with 0, 10, 20, 35, 50, and 100 μmol/L of suramin in DMEM without FBS. Migration of the wounded cells was evaluated after 0, 12, 24, and 48 hours with an inverted OLYMPUS CKX41 microscope.

### 2.9 In vitro invasion assays

Corning Biocoat Matrigel Invasion Chamber (Corning, Two Oak Park, MA, USA) was used for the invasion assays. HSC‐3 and SAS cells were washed in PBS and then suspended in DMEM without FBS. The cells (1.0 × 10^5^) were added to the upper chamber. The lower chamber was filled with 0, 20, 50, and 100 μmol/L of suramin in DMEM without FBS. After incubation for 24 hours, the cells were fixed with 10% formaldehyde neutral buffer solution (Sigma) for 20 minutes at room temperature, before being stained with crystal violet (0.05% in distilled water) (Katayama Chemical, Osaka, Japan) for 10 minutes. The invading cells were counted using an inverted OLYMPUS CKX41 microscope at ×40 magnification.

### 2.10 Statistics

Statistical analyses of significance were performed using unpaired Student's *t* test for comparison between two groups (control vs suramin treatment). The results are shown as the mean ± SD; *P* < 0.05 was considered statistically significant.

## 3 RESULTS

### 3.1 Screening of the approved drug library for targeting of HuR by DSF

**Figure 1:**
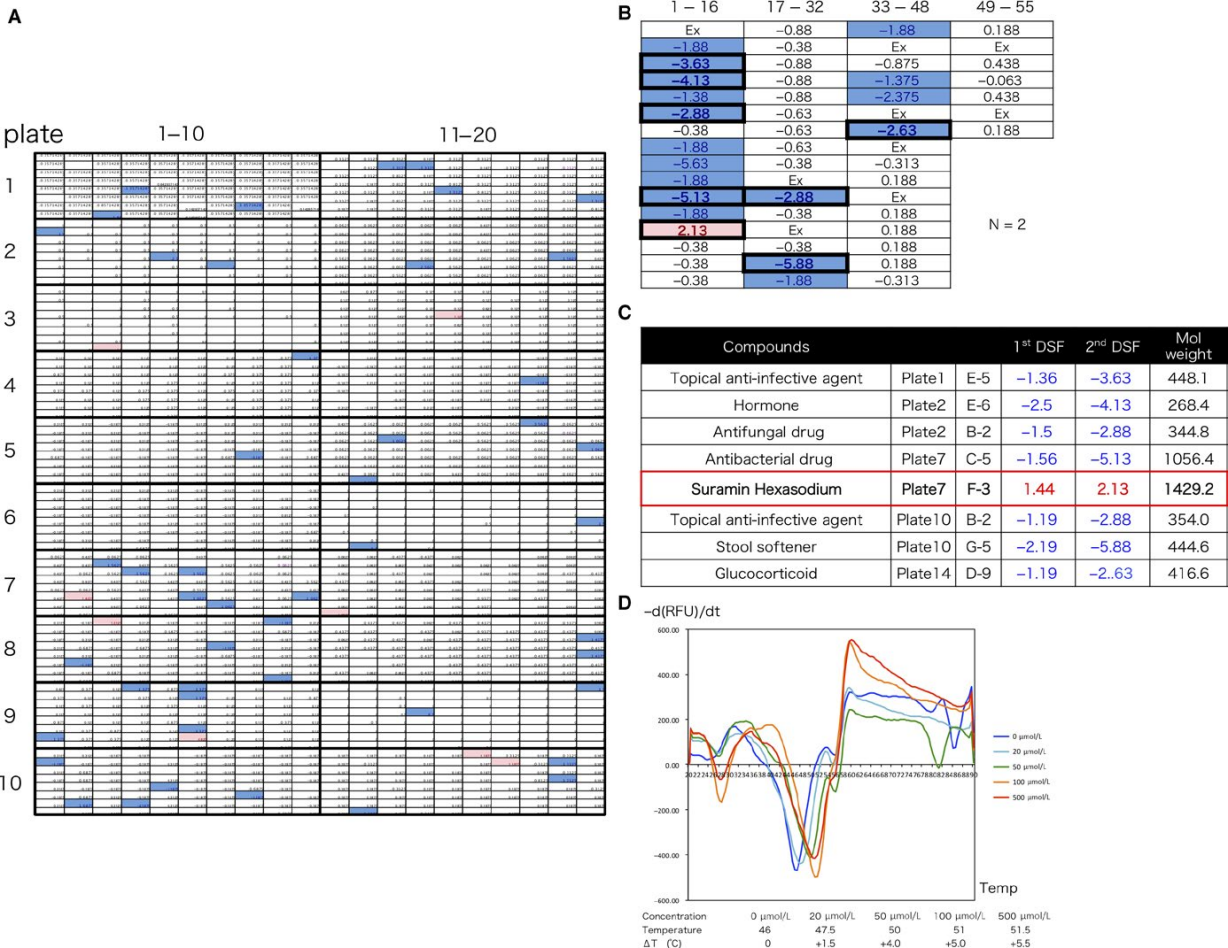
Screening of approved drug library for HuR targets by DSF. A, Screening of 1570 compounds in the approved drug library by DSF. The compounds were used at 200 μmol/L for HuR (5 μg) in the 1st DSF. Red boxes show *T*_m_ increase by more than 1.0°C. Blue boxes show *T*_m_ decrease by less than 1.0°C. A total of 80 compounds were placed in one plate. First DSF selected 55 compounds. B, Concentration‐dependent thermal shift was confirmed for 55 compounds. The compounds were used at 467 μmol/L for HuR (5 μg) in the 2nd DSF. The 2nd DSF selected eight compounds. Exclusion (Ex) of irregular waveform. C, Only suramin was shifted positively. All other compounds were shifted negatively. D, DSF for each concentration of suramin

To identify HuR‐interacting compounds, we screened 1570 compounds in the approved drug library by DSF. The compounds were used at 200 μmol/L for HuR (5 μg) in the first DSF. The *T*_m_ of 55 compounds was shifted by more than 1.0°C positively or negatively (Figure 1A). The concentration‐dependent thermal shift of *T*_m_ was confirmed for 55 compounds. The compounds were used at 467 μmol/L for HuR (5 μg) in the second DSF. The shift in the *T*_m_ of eight compounds was more than that in the first DSF (2.0°C positively or negatively; Figure 1B). Eight compounds of irregular waveform were excluded. Only suramin showed a positive shift; all other compounds (antibiotics, surface acting agent, hormone, etc) showed a negative shift (Figure 1C). Furthermore, to confirm HuR interaction of suramin, DSF was performed at each concentration of suramin. The difference in *T*_m_ was the highest from 20 μmol/L to 50 μmol/L, and the *T*_m_ at 100 and 500 μmol/L was almost the same (Figure 1D). These data show that suramin interacted with HuR in a concentration‐dependent manner.

### 3.2 Suramin binds to HuR on SPR

**Figure 2:**
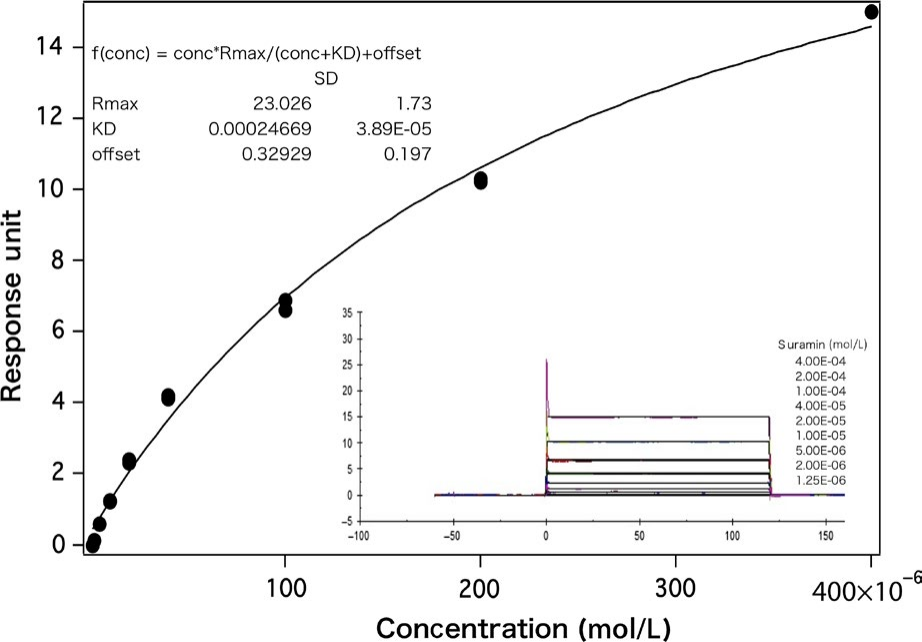
Suramin binds to HuR as shown by SPR. The binding interaction was concentration‐dependent. The KD value (2.4 × 10^−4^ mol/L) was obtained from the steady‐state phase data of the response versus concentration plot for nine different samples. The sensorgram showed square wave appearance exhibiting fast‐on and fast‐off kinetic behavior

SPRis label‐free and enables real‐time quantification of ligand‐binding affinities and kinetics.^28^ We performed SPR to evaluate the ligand‐binding affinities and kinetics between suramin and HuR. The SPR data are shown in Figure 2. The binding interaction between suramin and HuR was concentration‐dependent. The KD value (2.4 × 10^−4^ mol/L) was obtained from the steady‐state phase data. The sensorgrams showed square wave appearance, which shows that the interactions reached a steady state rapidly upon injection and also showed rapid dissociation from the binding site. Moreover, the sensorgrams exhibited fast‐on and fast‐off kinetic behavior.^29^ These results show that suramin binds to HuR, but with low affinity.

### 3.3 Suramin inhibits HuR functions

**Figure 3:**
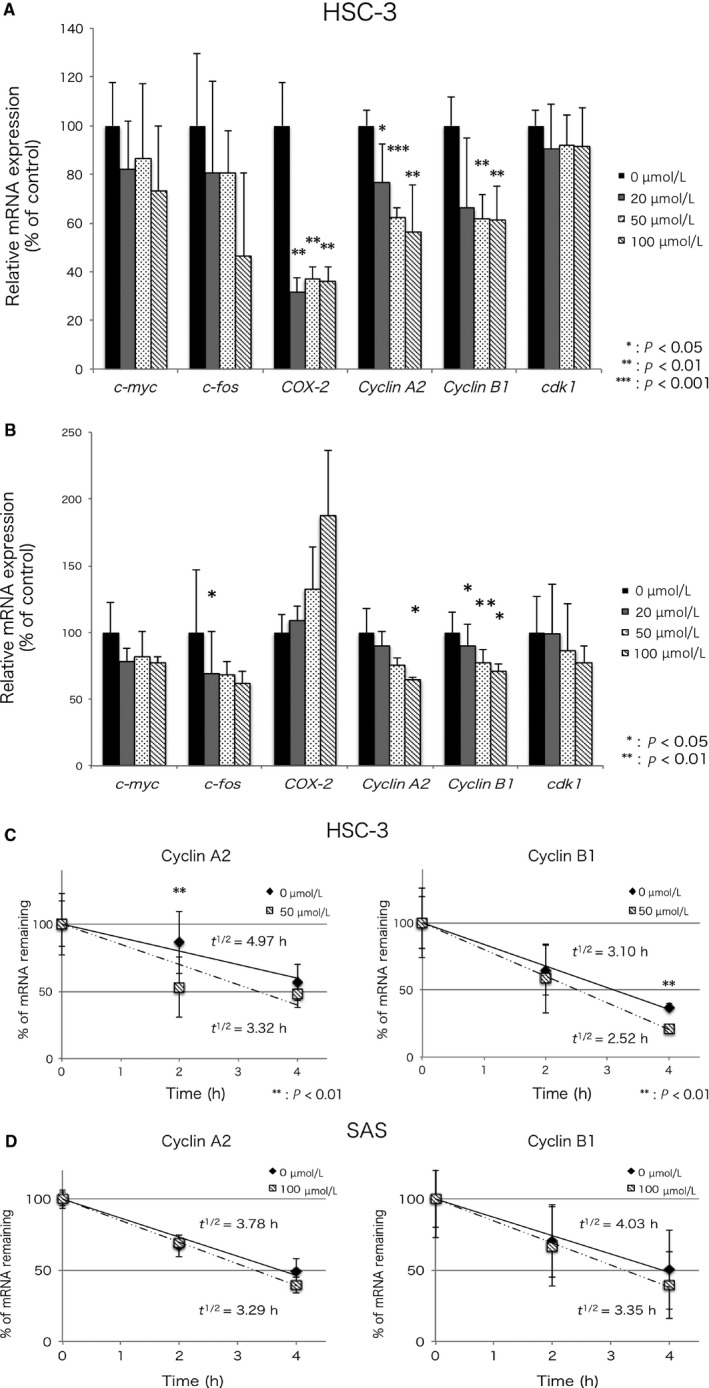
Suramin decreased the expression and stabilization of HuR‐binding mRNA. A,B, The accumulation of *c‐fos, c‐myc, COX‐2, cyclin A2, cyclin B1*, and *cdk1* was estimated by qRT‐PCR. The data represent the mean of four independent experiments in HSC‐3 cells and three independent experiments in SAS cells. C,D, Stabilization of ARE‐mRNA. The amount of each ARE‐mRNA was estimated at the indicated actinomycin D treated time point by qRT‐PCR. The *t*_1/2_ value indicates the half‐life of mRNA. The data represent the mean of three independent experiments. Error bars, SD. Student's *t* test, *P < 0.05, **P < 0.01, ***P < 0.001

HuR knockdown decreases the expression and stabilization of ARE‐mRNA such as*c‐fos*,* c‐myc*, and *COX‐2*, and mRNAs not containing ARE such as *cdk1* and *c‐fms*.^7^, ^17^, ^30^ The expression and stabilization of these mRNAs decreased when suramin inhibited HuR function. To confirm the expression of ARE‐mRNAs such as *c‐fos*,* c‐myc*,* COX‐2*,* cyclin A2*, and *cyclin B1*, and mRNAs not containing ARE such as *cdk1*, we performed qRT‐PCR on HSC‐3 and SAS cells treated with different concentrations of suramin. The expression of all mRNAs decreased in HSC‐3 cells. A significant decrease was observed in the expression of *COX‐2*,* cyclin A2*, and *cyclin B1*. The expression of cdk1 showed a slight decrease (Figure 3A). Similarly, the expression of all mRNAs except *COX‐2* decreased in SAS cells. A significant decrease was observed in the expression of *c‐fos*,* cyclin A2*, and *cyclin B1*. However, the expression of *COX‐2* increased in SAS cells (Figure 3B). Suramin did not decrease the binding of all mRNA to HuR. It showed different effect in different cell lines, such as on *COX‐2* levels. Furthermore, to confirm the stabilization of ARE‐mRNA, we determined the half‐life (*t*_1/2_) of *cyclin A2* and *cyclin B1* mRNA in the control‐ and suramin‐treated cells. The expression of *cyclin A2* and *cyclin B1* mRNA was estimated at 0, 2, and 4 hours after treatment with actinomycin D (an inhibitor of RNA polymerase II) by qRT‐PCR, and the half‐life of these mRNAs was calculated (Figure 3C,D). In suramin‐treated HSC‐3 cells, the half‐life of *cyclin A2* and *cyclin B1* mRNA was 3.32 and 2.52 hours, respectively, whereas in the control cells, the half‐life was 4.97 and 3.10 hours, respectively (Figure 3C). In suramin‐treated SAS cells, the half‐life of *cyclin A2* and *cyclin B1* mRNA was 3.78 and 3.29 hours, whereas in the control cells, the half‐life was 4.03 and 3.35 hours, respectively (Figure 3D). To confirm that HuR overexpression inhibits decrease in expression of ARE‐mRNA for treatment of suramin, we performed to transfection in the HSC‐3 cells with plasmid of pcDNA3 or pcDNA3‐HuR and treatment of difference concentration of suramin. Though it is no significant difference, decrease rate of expression of the *cyclin A2* and *cyclin B1* mRNA in the suramin‐treated cells for the control cells was smaller in the cells transfected pcDNA3‐HuR than in the cells transfected control plasmid (Figure S2). Next, to confirm the location of HuR, we performed immunofluorescence staining. Suramin did not change the localization of HuR (Figure S3). These data indicate that suramin decreases the expression and stabilization of ARE‐mRNA but does not affect nucleo‐cytoplasmic transport, suggesting that suramin inhibits HuR functions, such as mRNA binding.

### 3.4 Suramin decreased the levels of proteins translated from ARE‐mRNA

**Figure 4:**
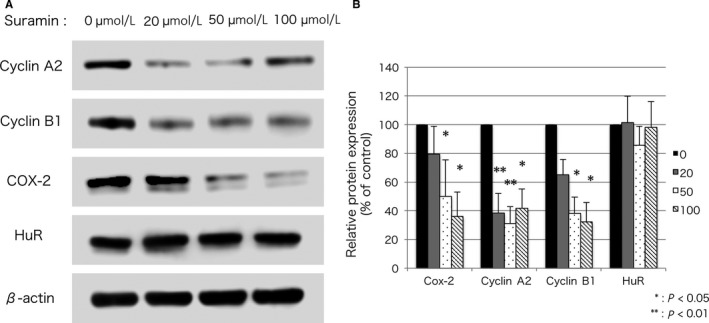
Suramin decreased the levels of proteins translated from ARE‐mRNA. A, HSC‐3 cells were treated with or without suramin, and the expression of COX‐2, cyclin A, cyclin B1, and HuR was estimated by Western blotting. B, Results of densitometry. Error bars, SD. Student's *t* test, *P < 0.05, **P < 0.01

We performed Western blotting to confirm whether the levels of proteins translated from ARE‐mRNA decreased in suramin‐treated HSC‐3 cells. COX2, cyclin A2, and cyclin B1 protein levels decreased in suramin‐treated cells compared with the control cells (Figure 4). Suramin did not decrease the HuR protein level (Figure 4). These results indicate that suramin decreased COX‐2, cyclin A2, and cyclin B1 protein levels to inhibit HuR functions, such as mRNA stabilization.

### 3.5 Suramin decreases cell viability in a concentration‐dependent manner

**Figure 5:**
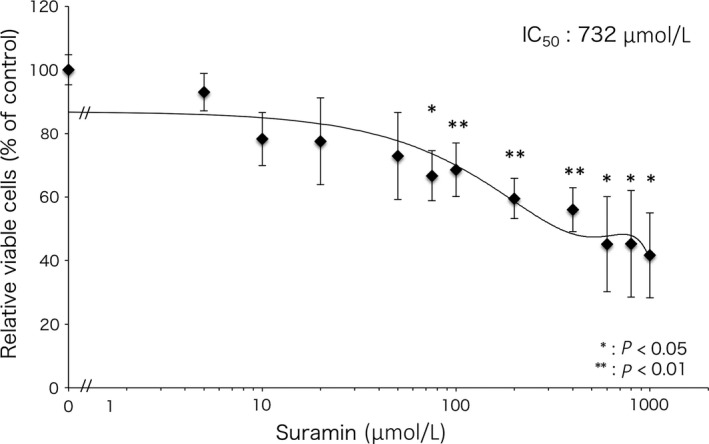
Survival of HSC‐3 cells treated with suramin by MTS assay. The survival rate of HSC‐3 cells treated with suramin was assessed by MTS assay. The concentration dependence of suramin decreased the survival rate of HSC‐3 cells. The IC_50_ value of suramin is 732 µmol/L. The data represent the mean of three independent experiments. Error bars, SD. Student's *t* test, *P < 0.05, **P < 0.01

MTS assay was performed to confirm the cytotoxicity of suramin against HSC‐3 cells. Although suramin decreased the viability of HSC‐3 cells in a concentration‐dependent manner, it showed low cytotoxicity (IC_50_ of suramin = 732 µmol/L). The cell viability at the highest treated concentration of suramin (1000 µmol/L) was 42% (Figure 5). These results suggest that suramin exhibited low cytotoxicity and did not induce necrosis strongly.

### 3.6 Suramin attenuated motile and invasive activities of HSC‐3 and SAS cell line

**Figure 6:**
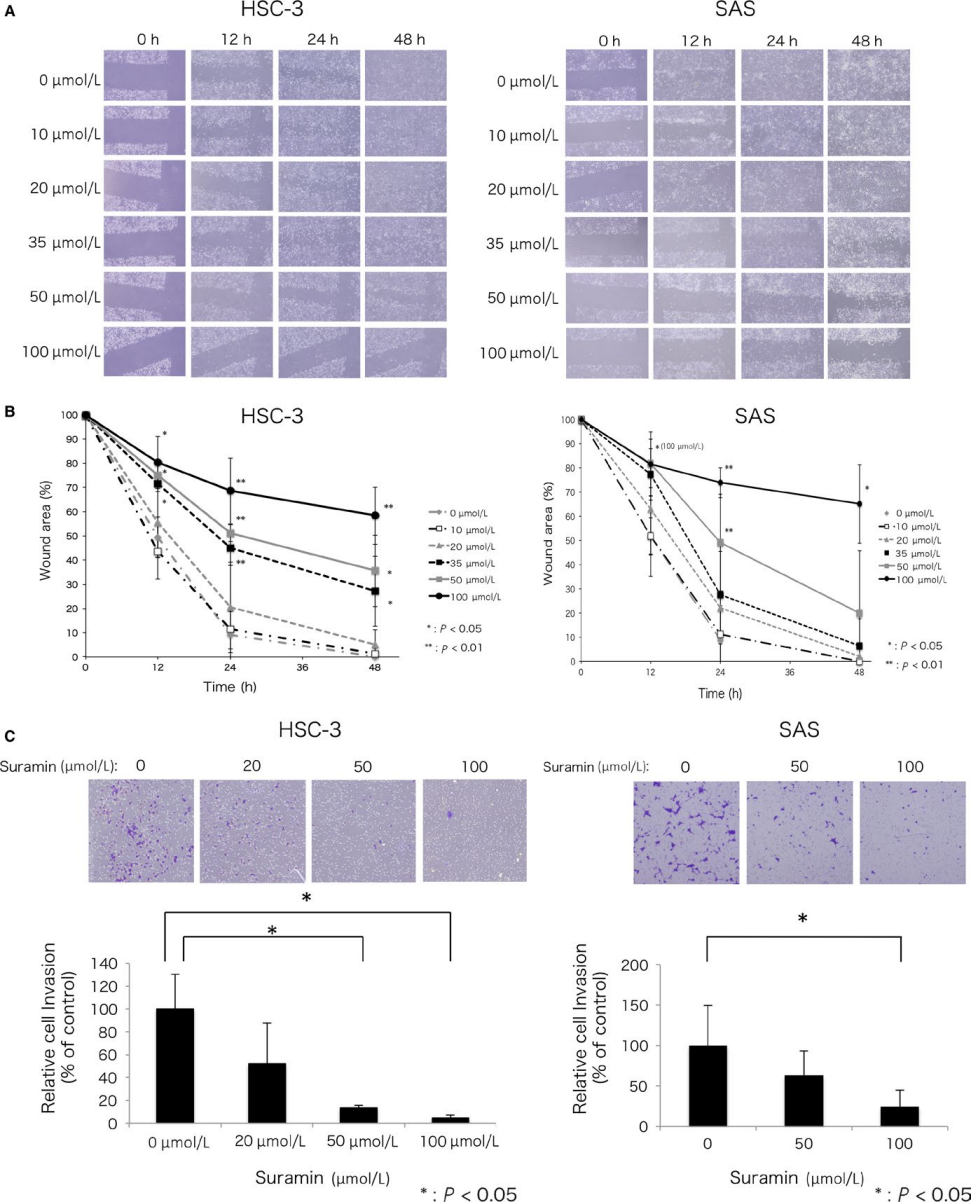
Suramin attenuated motility and invasion. A, Phase images of HSC‐3 and SAS cells treated with different concentrations of suramin at 0, 12, 24, and 48 h after wounding. B, Quantification of cell motility analyzed by wound healing assay. The data indicate the mean of three independent experiments. C, Invasion rate of HSC‐3 and SAS cells treated with different concentrations of suramin by using the Corning Biocoat Matrigel Invasion Chamber. After 24 h, the cells on the lower side of the membrane were fixed, stained, and counted. The data represent the mean of three independent experiments. Error bars, SD. Student's *t* test, * *P* < 0.05, **P < 0.01

In our previous report, we showed that HuR knockdown attenuates motile and invasive activities.^7^ We presumed that suramin attenuates the motile and invasive activities of HSC‐3 and SAS cells as well as HuR knockdown HSC‐3 cells. To prove this, wound healing and invasion assays were performed. HSC‐3 and SAS cells treated with each concentration of suramin were scratched and observed after 12, 24, and 48 hours. At a concentration of 35 µmol/L or above, suramin suppressed the motile activity of HSC‐3 cells significantly (*P* < 0.05 or 0.01; Figure 6A,B). At a concentration of 50 µmol/L or above, suramin suppressed the motile activity of SAS cells significantly (*P* < 0.05 or 0.01; Figure 6A,B). Next, HSC‐3 and SAS cells were seeded in invasion chambers containing Matrigel‐coated membranes, and the lower chamber was filled with different concentrations of suramin. Treatment of HSC‐3 cells with suramin at concentrations above 50 µmol/L strongly suppressed the invasive activity compared to control cells (*P* < 0.05; Figure 6C). Suramin (20 µmol/L) tended to suppress the invasive activity of HSC‐3 cells (*P* = 0.051). Treatment of SAS cells with suramin at 100 µmol/L strongly suppressed the invasive activity as compared to control cells (*P* < 0.05; Figure 6C). These results suggest that suramin strongly attenuated the motile and invasive activities and thus the malignant phenotype of HSC‐3 and SAS cells.

## 4 DISCUSSION

In this study, we screened 1570 compounds in the approved drug library by DSF and selected suramin as a potential HuR‐targeted anticancer drug. Suramin exhibited anticancer effects by inhibiting HuR function in HSC‐3 cells. HuR binds and stabilizes the ARE‐mRNA‐related malignant phenotype, such as proliferation, invasion, and metastasis.^3^ In our previous studies,^7^, ^17^ we showed that HuR was expressed abundantly in oral cancer cells, and HuR knockdown attenuated the malignant phenotype of these cells. Therefore, HuR‐targeted compounds that inhibit HuR functions such as ARE‐mRNA stabilization can be used as novel anticancer agents. Recently, certain HuR inhibitors were reported, such as MS‐444,^13^ quercetin,^27^ azaphilone‐9,^24^ and some other small molecule compounds.^14^, ^25^ As HuR inhibitors are still in the initial stages of development, it will take a long time to reach the clinical trial stage.

Drug development is time‐consuming and expensive. However, repositioning of approved drugs can function more efficiently and minimize the costs and risks compared to experimental drug development from the start.^26^ Pharmakon‐1600 is an approved drug library, which combines US drug collection and international drug collection. The US drug collection comprised FDA approved drugs. The international drug collection is used in Europe and Asia, but not approved for use in the United States. Therefore, we chose the approved drug library to screen compounds.

We used DSF for identifying HuR‐interacting compounds in the library. DSF is a high‐throughput and inexpensive screening method to identify low‐molecular‐weight ligands that bind and stabilize purified proteins.^31^ In the first and second DSF, eight compounds were selected from 1570 compounds (Figure 1A,B). Only suramin shifted the *T*_m_ positively in a concentration‐dependent manner (Figure 1C,D). In most cases, if a compound binds to a protein, the free energy contribution of ligand‐binding results in an increase in Gibbs free energy of unfolding, and this might cause an increase in *T*_m_. The stabilizing effect of compounds on binding is proportional to the concentration and affinity of the ligands.^31^ The results of DSF suggest that suramin interacts and binds to HuR. We performed SPR to confirm the affinities and kinetics between suramin and HuR. As shown by SPR, suramin binds to HuR, exhibiting fast‐on and fast‐off kinetic behavior. Therefore, we selected suramin as a potential HuR inhibitor.

Suramin is a polysulfonated naphthylurea introduced in the 1920s for the treatment of African trypanosomiasis and onchocerciasis by inhibiting DNA polymerases and reverse transcriptase. At the end of the 1970s, it was found to competitively inhibit retroviral reverse transcriptase.^32^ Since the late 1980s, the activity of suramin as an anticancer agent in hormone‐refractory prostate cancer (HRPC) patients has been appreciated.^33^, ^34^ Suramin exerts antitrypanosomal, anticancer, and antiviral activities.^32^, ^33^, ^34^, ^35^ It is capable of blocking the binding of growth factors, such as EGF, TGF‐β, PDGF, bFGF, and VEGF, to their surface receptors.^33^, ^36^, ^37^, ^38^ In addition, suramin decreases the expression of cell proliferation markers such as cyclin A, cyclin D1, and cyclin E, arrests cells in G1, and decreases the number of cells in S‐phase moderately.^35^ However, the degree to which the anticancer effects of suramin are mediated by the above mechanisms is unclear. We presumed that the anticancer effects of suramin are caused by the inhibition of HuR function and confirmed that suramin decreases the expression and stabilization of HuR‐binding mRNA (Figure 3A). *Cdk1* expression was slightly decreased compared to that of other mRNAs in HSC‐3 cells. *COX‐2* expression significantly decreased in HSC‐3 cells, but increased in SAS cells. Similarly, suramin treatment showed different effects on ARE‐mRNA in different cell lines.

We found that suramin decreased the protein levels of COX‐2, cyclin A2, and cyclin B1 in HSC‐3 cells (Figure 4). It affected the growth factors and cyclin‐related proteins^33^, ^35^, ^36^, ^37^, ^38^ that translated from HuR‐binding ARE‐mRNA.^3^ HuR knockdown led to G1 arrest^39^ in cancer cells as well as suramin‐treated cells,^35^ because HuR regulates cell cycle through stabilization of cell cycle‐related ARE‐mRNA.^40^, ^41^ These reports suggested the association between suramin and HuR. Recently, it was reported that suramin improved language and social interaction, and decreased restricted or repetitive behaviors of autism spectrum disorder (ASD).^42^ However, the mechanism underlying the effects of suramin in ASD is unclear. HuR regulated the translation of mRNA transcribed from autism‐associated genes, *Foxp1* and *Foxp2*.^43^ Suramin may have improved ASD by inhibiting HuR‐*Foxp1* and/or HuR‐*Foxp2* binding. Thus, from these findings, it can be suggested that suramin inhibits HuR functions.

Noncytotoxic concentration of suramin was found to be less than 50 µmol/L, whereas it showed high cytotoxicity at concentrations more than 200 µmol/L.^44^ Effective plasma concentration of suramin was reported to be between 100 and 200 µmol/L.^45^ Therefore, MTS, wound healing, and invasion assays were performed to find the concentration of suramin that affects the malignant phenotype of oral cancer cells. In MTS assay, suramin did not induce cell death strongly at low‐moderate concentrations (<100 µmol/L). However, low‐moderate concentrations of suramin (50 and 100 µmol/L) attenuated motile and invasive activities markedly in HSC‐3 cells with high metastatic potential. Suramin at 100 µmol/L attenuated these activities in SAS cells. These results showed that low‐moderate concentration of suramin exhibits low cytotoxicity, but affects motile and invasive activities strongly in oral cancer cells.

Large clinical trials of suramin for cancer have been reported previously. A double‐blind placebo‐controlled phase III trial was undertaken to evaluate the efficacy of suramin plus hydrocortisone (HC) in patients with symptomatic HRPC.^34^ The adrenal suppressive properties of suramin require the concomitant administration of HC. Compared to patients who received placebo plus HC, those who received suramin plus HC showed delayed disease progression, decline in prostate‐specific antigen (PSA), and effective reduction of pain. In the trials, most adverse events were mild or moderate intensity and easily managed medically. The suppression of disease progression and reduction of pain can be explained by the decrease in cyclin‐related and COX‐2 protein levels in HSC‐3 cells by suramin. Although suramin has not been used for the treatment of oral cancer, our results suggest that suramin can suppress disease progression and invasion and may reduce pain by decreasing COX‐2 protein levels depending on cell line. Oral cancer leads to poor quality of life due to intense pain and significantly impairs speech, swallowing, and masticatory functions.^46^ Oral cancer pain management is difficult. Therefore, suramin, which reduces pain, can be used effectively for oral cancer therapy.

Our study had some limitation. Firstly, higher affinity is ideally preferred for an HuR inhibitor; however, suramin binds to HuR with low affinity (KD value 2.4 × 10^−4^ mol/L). Secondly, cells were treated with suramin in the absence of serum to exclude the effect of other serum components. Thirdly, suramin has been reported to have anticancer effects in vivo, but we could not report whether suramin could inhibit HuR function in vivo.

In conclusion, in this report, we show that suramin binds to HuR and inhibits HuR functions such as ARE‐mRNA stabilization and translation. In addition, suramin markedly attenuates the malignant phenotype of HSC‐3 and SAS cells. Therefore, suramin, a HuR‐targeted drug, has potential as a new therapeutic agent for oral cancer therapy. Our results suggest that suramin, via its different mechanism, may effectively suppress progressive oral cancer that cannot be controlled using other anticancer agents.

## CONFLICT OF INTEREST

None declared.
