# Supplementary figures and images for: Suramin, screened from an approved drug library, inhibits HuR functions and attenuates malignant phenotype of oral cancer cells
Source: Cancer Med. 2018 Nov 18;7(12):6269–80. doi: 10.1002/cam4.1877 (PMC6308099; doi:10.1002/cam4.1877)

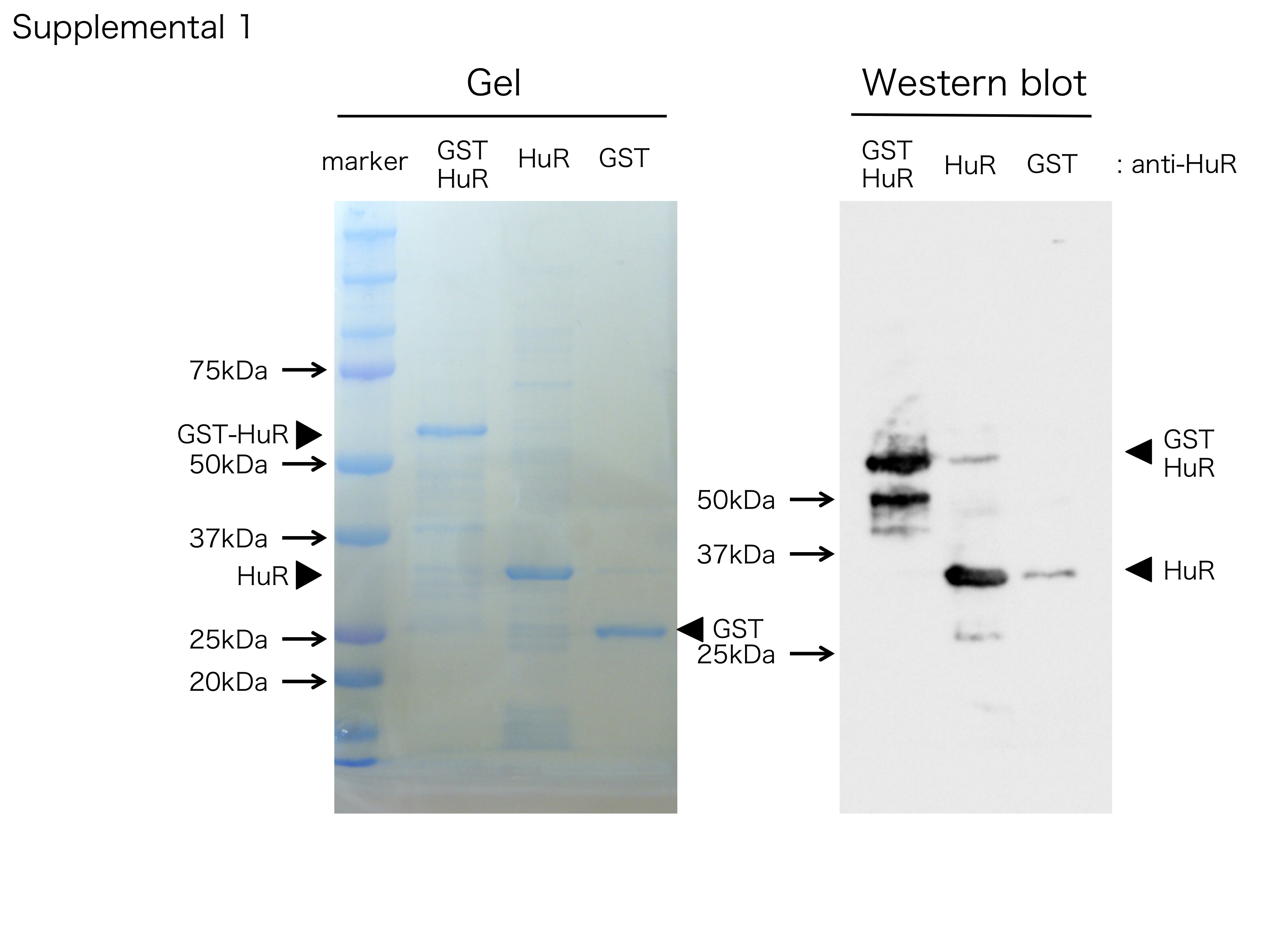

Supplement: Supplementary file 1 [file CAM4-7-6269-s001.tiff]

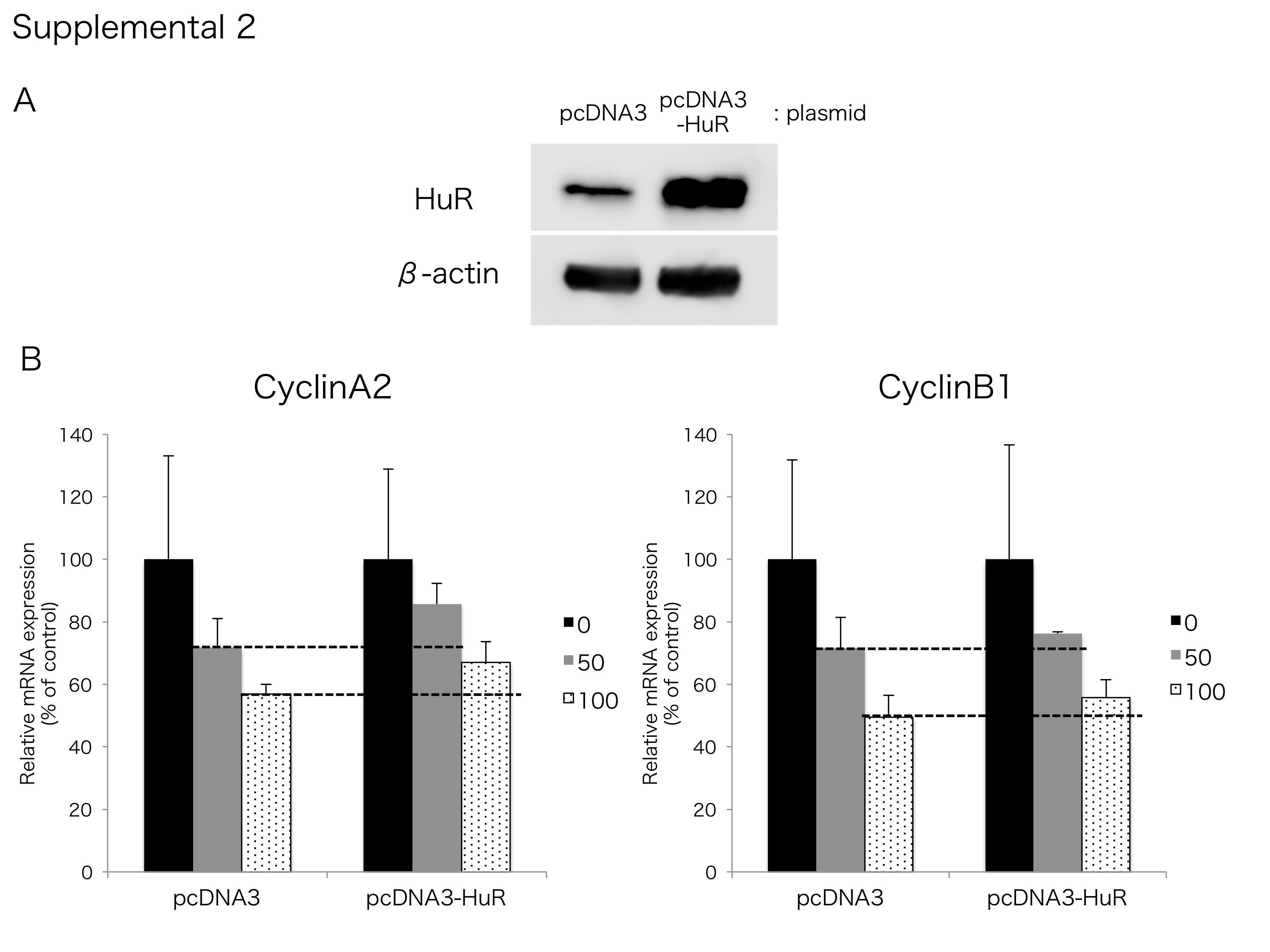

Supplement: Supplementary file 2 [file CAM4-7-6269-s002.tiff]

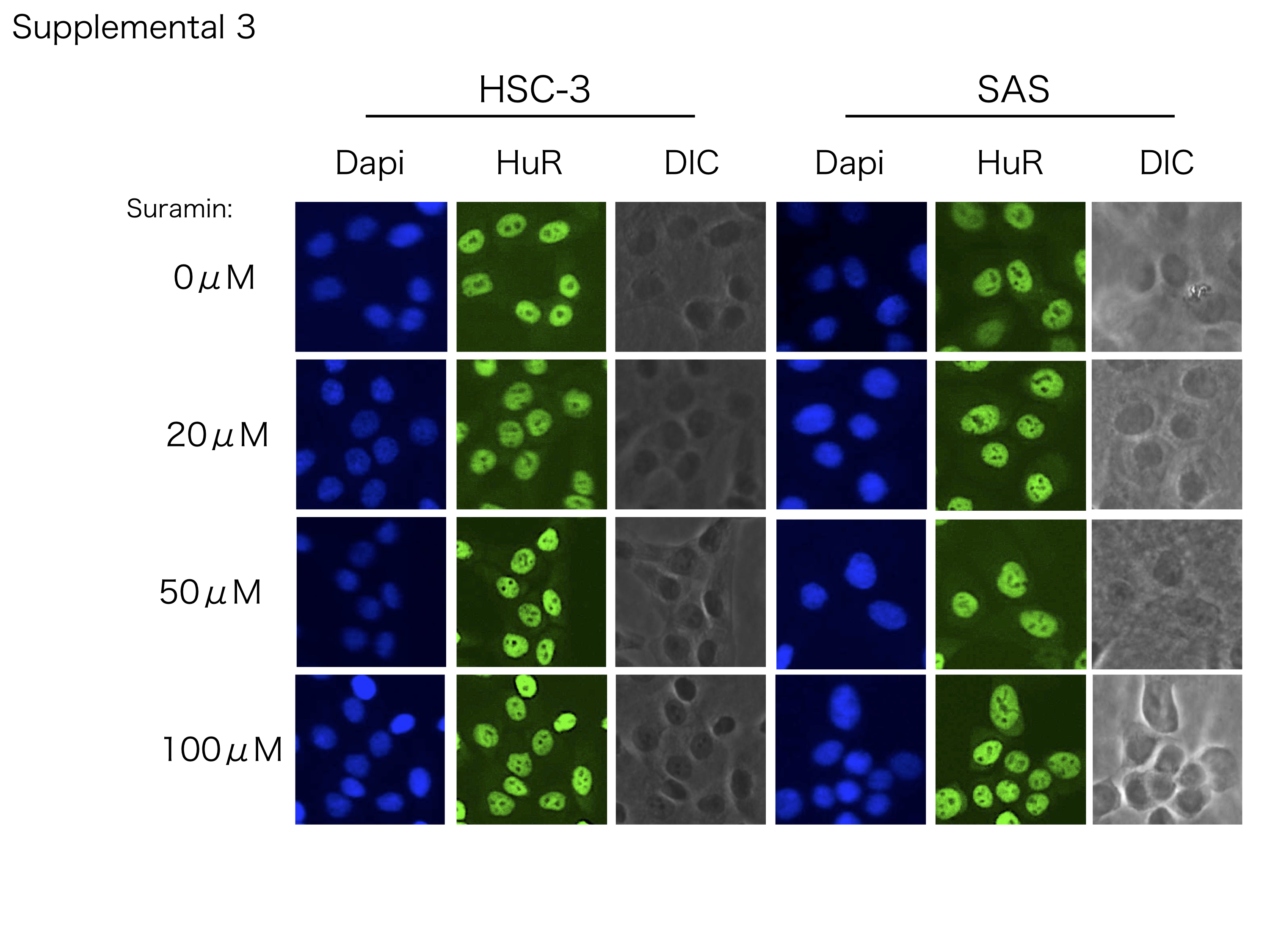

Supplement: Supplementary file 3 [file CAM4-7-6269-s003.tiff]
