# Supplementary material for: Suramin, screened from an approved drug library, inhibits HuR functions and attenuates malignant phenotype of oral cancer cells
Source: Cancer Med. 2018 Nov 18;7(12):6269–80. doi: 10.1002/cam4.1877 (PMC6308099; doi:10.1002/cam4.1877)
Supplement: Supplementary file 4 [file CAM4-7-6269-s004.docx]

**Supplemental data**

**MATERIALS and METHODS**

*Gel stain*

GST-HuR, HuR, and GST protein were separated on 10% TGX FastCast^TM^ acrylamide gel (Bio-Rad). Then, the gel was stained with One-step CBB stain solution (BIO CRAFT, Tokyo, Japan).

*Transfection of plasmid*

HSC-3 cells (1.5 × 10^5^) were harvested in DMEM containing 10% FBS without antibodies in 6-well plates. After 24 h, the culture medium was changed and the cells were transfected with TransFectin^TM^ Lipid Reagent (Bio-rad). For transfection, Opti-MEM^TM^ (Thermo Fisher) mixed with 3µg of pcDNA3 vectors (pcDNA3) or pcDNA3 containing sequences coding for full-length HuR (pcDNA-HuR) and 6µl of TransFectin was added to the medium. After 24 h, the cells treated for 24h with 0, 50, and 100µM of suramin in DMEM without FBS and antibodies. Then, Western blot and qRT-PCR were performed on the cells.

*Immunofluorescence staining*

HSC-3 and SAS cells were harvested in Eppendorf Cell Imaging Slides. The cells were treated with different concentrations (0, 20, 50, and 100 μM) of suramin for 24 h in DMEM without FBS. Then, the cells were fixed with 10% formaldehyde neutral buffer solution (Sigma) for 20 min at room temperature and permeabilized with 0.5% Triton X-100 (Sigma) for 10 minutes on ice. The cells were blocked using PBS with 1% Tween 20 (Sigma) and 0.1% BSA (Sigma). The primary antibody was specific to HuR (sc-5261, 1:500, Santa Cruz) and the secondary antibody was Alexa Fluor^TM^ 488 F(ab’)2 fragment goat anti-mouse IgG(H+L) (1:500, Invitrogen). The cells were mounted using ProLong^TM^ Diamond Antifade Mountant with DAPI (Invitrogen). The fluorescence and phase images were acquired by FLUOVIEW FV10i (OLYMPUS).

Supplemental Fig. 1. HuR protein was confirmed to perform gel stain and western bolt.

(A) Recombinant GST-HuR, HuR, and GST protein bands were detected with CBB staining. (B) Western blot was performed on recombinant GST-HuR, HuR, and GST proteins.

Supplemental Fig. 2. HuR overexpression inhibits decrease in expression of ARE-mRNA for treatment of suramin.

(A) HSC-3 cells were transfected with pcDNA3 or pcDNA3-HuR plasmid. HuR was overexpressed in the transfected pcDNA3-HuR than control vector (pcDNA3). (B) HSC-3 cells were treated with or without suramin, and the expression of *cyclin A2* and *cyclin B1* was estimated by qRT-PCR. Decrease rate of expression of the mRNA in the suramin treated cells for the control cells (0µM of suramin) was smaller in the cells transfected pcDNA3-HuR than in the cells transfected control plasmid. The data represent the mean of three independent experiments. Error bars, SD. No significant difference.

Supplemental Fig. 3. Suramin does not change HuR localization.

The fluorescence and phase images were acquired for both HSC-3 and SAS cells treated with different concentrations of suramin. DAPI fluorescence is shown in blue, whereas HuR is represented by green. The results presented are representative results of one of three independent experiments.
